# Individual socioeconomic position, neighbourhood disadvantage and mental well-being: a cross-sectional multilevel analysis of mid-age adults

**DOI:** 10.1186/s12889-022-12905-7

**Published:** 2022-03-14

**Authors:** Emily M. Mann, Kristiann C. Heesch, Jerome N. Rachele, Nicola W. Burton, Gavin Turrell

**Affiliations:** 1 School of Public Health and Social Work, Queensland University of Technology, Victoria Park Road, Kelvin Grove QLD, Brisbane, Queensland 4059 Australia; 2 College of Health and Biomedicine and Institute for Health and Sport, Victoria University, Footscray, Victoria Australia; 3 School of Applied Psychology, Griffith University, Brisbane, Queensland Australia; 4 Menzies Health Institute Queensland, Griffith University, Brisbane, Queensland Australia; 5 School of Global, Urban and Social Studies, RMIT University, Melbourne, Victoria Australia

**Keywords:** Socioeconomic inequality, Neighbourhood disadvantage, Mental well-being, Multilevel analysis

## Abstract

**Background:**

Socioeconomic disadvantage is associated with mental illness, yet its relationship with mental well-being is unclear. Mental well-being is defined as feeling good and functioning well. Benefits of mental well-being include reduced mortality, improved immune functioning and pain tolerance, and increased physical function, pro-social behaviour, and academic and job performance. This study aims to explore the relationship between individual socioeconomic position (SEP), neighbourhood disadvantage and mental well-being in mid-age adults.

**Methods:**

Multilevel modelling was used to analyse data collected from 7866 participants from the second (2009) wave of HABITAT (How Areas in Brisbane Influence healTh and activiTy), a longitudinal study (2007–2018) of adults aged 40–65 years living in Brisbane, Australia. Mental well-being was measured using the Warwick Edinburgh Mental Well-Being Scale (WEMWBS). Exposure measures were education, occupation, household income, and neighbourhood socioeconomic disadvantage.

**Results:**

The lowest MWB scores were observed for the least educated (β = − 1.22, 95%CI = − 1.74, − 0.71), those permanently unable to work (β = − 5.50, 95%CI = − 6.90, − 4.10), the unemployed (β = − 2.62, 95%CI = − 4.12, − 1.13), and members of low-income households (β = − 3.77, 95%CI = − 4.59, − 2.94). Residents of the most disadvantaged neighbourhoods had lower MWB scores than those living in the least disadvantaged neighbourhoods, after adjustment for individual-level SEP (β = − 0.96, 95%CI = − 1.66, − 0.28).

**Conclusions:**

Both individual-level SEP and neighbourhood disadvantage are associated with mental well-being although the association is stronger for individual-level SEP. This research highlights the need to address individual and neighbourhood-level socioeconomic determinants of mental well-being.

**Supplementary Information:**

The online version contains supplementary material available at 10.1186/s12889-022-12905-7.

## Background

During the last decade, a growing number of studies have examined the relationship between individual-level socioeconomic position (SEP), neighbourhood disadvantage and mental health. With few exceptions, [1] most of this work shows that people of low SEP [2] or those living in disadvantaged neighbourhoods (after adjustment for individual level covariates) experience poorer mental health [3]. Most studies of mental health and SEP conceptualise mental health as being synonymous with a health problem that significantly affects how a person feels, thinks, behaves, and interacts with other people: [4] for example, psychological distress, anxiety, and depression. Notwithstanding the important contribution of these studies, mental health is recognised as being more than the absence of psychopathology; it is combined states of health and illness with two distinct, but correlated, unipolar continua [5]: (1) mental illness or disorder, and (2) mental well-being (MWB).

MWB comprises two dimensions, namely how we feel (the subjective experience of positive emotions) and how we function (psychological functioning, good relationships with others, and self-realisation) [6]. Characterised by a U-shape trajectory over the life-course, MWB is high in early life, dips in mid-life, and increases again in older age [7], when it may be most beneficial to health. Health outcomes of higher MWB include reduced mortality [8]; increased immune system function, pain tolerance [9] and physical function [10]; and improved health optimism [11] and pro-social behaviour [12]. Some of these health outcomes are evidenced by studies of psychobiological systems linking positive affect with distinct biological correlates (favourable associations with heart rate, blood pressure, and inflammatory markers such as interleukin-6) [13]. These results are independent of negative affect and depressed mood [14], further supporting the dual continua model of mental health.

To date, few studies have examined associations between individual-level SEP, neighbourhood disadvantage, and MWB. Studies that have investigated associations between individual-level socioeconomic factors and MWB, measured with the Warwick-Edinburgh Mental Well-being Scale (WEMWBS), have shown that people with low SEP have increased risk of low MWB, regardless of the SEP measure used [6, 15]. The aetiology of this relationship is complex, and although SEP measures are interrelated, they each reflect different aspects of SEP and act through direct and indirect pathways [16]. It is reasonable to hypothesise that the pathways between SEP and MWB—as distinct from those between SEP and mental illness—are mediated by a set of factors that promote flourishing. Education may promote MWB by enhancing problem-solving abilities and acquisition of positive social, psychological (self-esteem and self-efficacy) and economic skills and assets [17]. Occupation may contribute to MWB via characteristics of work and workplace, including control, variety and use of skills, social support, work pace and job satisfaction [18]. Income reflects economic and material resources that provide opportunities to reside in quality housing and neighbourhoods (which are associated with positive psychosocial environments) [19] and the means to partake in activities (e.g., volunteer work and engage in leisure activities), [20] which could contribute to MWB.

Research spanning the last 25 years consistently shows that neighbourhood disadvantage—characterised by poverty, deprivation, and social and economic disadvantage brought about by an area’s social, cultural, and economic factors and its physical and environmental infrastructure [21]—adversely influences health, independent of individual-level SEP. [22] Neighbourhood disadvantage may also adversely influence MWB via lack of reciprocity and social connections (enabled through social networks and social support), [23] neighbourhood problems (e.g., noise, vandalism, traffic, and smells), [24] or perceptions of poor neighbourhood quality (e.g., unattractiveness of the environment) [19]. Many studies support an association, although modest, between neighbourhood disadvantage and mental illness, [25] yet the relationship between neighbourhood disadvantage and MWB is unclear. To date, two studies have examined the relationship between neighbourhood disadvantage and MWB. In a British study [24] no association between area-level deprivation and MWB was found. The lack of association may have been due to a low average level of area deprivation as the sample was from a socioeconomically advantaged county. Also, the sample was older, aged 69–78 years, and at older ages any differential socioeconomic association with health is reduced, partly due to those from lower SEP experiencing disproportionate rates of death at earlier ages [26, 27]. In a Northern Ireland study, McAnerny et al. [23] reported unadjusted relationships between MWB and strata of neighbourhood deprivation. A suggestive relationship was indicated by non-overlapping confidence intervals for the mean MWB scores in the least deprived neighbourhoods and other categories of deprivation (except the second most deprived).

Beyond examining the main effects of neighbourhood disadvantage and MWB, testing interactions between the same level of SEP and different levels of neighbourhood disadvantage can highlight ‘deprivation amplification’ effects [28] and help identify vulnerable neighbourhoods and population groups [29]. For example, individuals with a low education level and living in disadvantaged neighbourhoods might have worse MWB than individuals with a low education level and living in advantaged neighbourhoods because the latter group may benefit from the collective resources (e.g., social networks, attractive environment) missing from disadvantaged neighbourhoods [17]. Indeed, McAneney et al. examined interactions between education, unemployment, and income and neighbourhood disadvantage, yet found no association. However, associations between unemployment and MWB, but not education and income, differed significantly across five neighbourhood deprivation strata: unemployed participants in the least deprived neighbourhoods had MWB scores almost double those of unemployed participants living in the most deprived [23].

Given the accumulating evidence on the protective effects of MWB on physical health and the adverse mental health outcomes of low SEP, there is reason to further explore associations between socioeconomic characteristics and MWB at the individual- and neighbourhood-levels. The aims of this study were to investigate: (1) the relationships between individual-level SEP, neighbourhood disadvantage and MWB, and (2) whether the relationship between SEP and MWB differed across levels of neighbourhood disadvantage. We hypothesised that residing in disadvantaged neighbourhoods would be associated with lower MWB, independent of individual-level socioeconomic factors (which themselves would be related to MWB). If this reasoning is supported, policies and programs aimed at reducing socioeconomic inequality in MWB should target both individuals and their neighbourhood contexts.

## Methods

This study used data from the second (2009) wave of HABITAT (How Areas in Brisbane Influence healTh and ActTvity), a multilevel longitudinal study (2007–2018) of mid-age adults living in Brisbane, Australia. Consent to participate was obtained via return of the participants’ completed survey. Data for this study were analysed from June 2020 to January 2021. Methods were carried out in accordance with relevant guidelines and regulations; HABITAT received ethics approval from the Queensland University of Technology Human Research Ethics Committee (Ref. No. 3967H & 1,300,000,161).

### Study sample

HABITAT’s sampling design has been published elsewhere [30]. Briefly, a multi-stage probability sampling design was used to select a stratified random sample (*n* = 200) of Census Collector’s Districts (CCD) in 2007 [31]. CCDs were the smallest administrative units used by the Australian Bureau of Statistics (ABS) in 2007, containing an average of 200 private dwellings. A random sample of people aged 40–65 years from each neighbourhood were invited to participate (approximately 85 people per CCD).

Eligible study participants were mailed a self-administered survey between May and July in the years 2007, 2009, 2011, 2013 and 2016 using the method by Dillman [32]. Of 16,128 surveys mailed in 2007, valid responses were received from 11,035 (68.4% response rate). Respondents were representative of the 2006 Brisbane population, although residents from disadvantaged areas, blue-collar employees, and persons who did not attain a post-school educational qualification were underrepresented [33]. In 2009, 7866 (72.3%) eligible and contactable participants responded.

### Measures

Mental well-being, the outcome, was measured using the Warwick-Edinburgh Mental Wellbeing Scale (WEMWBS), which comprises items on subjective well-being, psychological functioning, and relationships [7]. The 2009 wave of HABITAT asked all 14 WEMWBS items. The 14-item WEMWBS scale has been well validated [7, 34] and used in the UK to monitor population-level well-being and evaluate interventions, policies, and programs aimed at improving mental wellbeing [7]. Responses to each item are scored on a 5-point Likert scale, ranging from ‘none of the time’ (1) to ‘all of the time’ (5), then summed to give a total score. The potential minimum and maximum scores are 14 and 70, respectively, with scores of 45–59 indicating average MWB and scores of 60 or more indicating high MWB [7]. In the current study, Cronbach’s alpha of the scale items was high at 0.96.

Socioeconomic predictor variables included education, occupation, household income and neighbourhood disadvantage.

#### Education

Participants selected their highest level of education attainment from nine response categories. These were recoded to bachelor’s degree or higher (including graduate certificate or diploma, Masters’ degree or doctorate), diploma or associate degree, certificate (trade or business) and no post-secondary school qualification.

#### Occupation

Employed respondents provided the full title of their occupation. This information was subsequently coded to the Australian and New Zealand Classification of Occupations (ANZCO) [35]. The original nine-level classification was recoded into three categories: managers/professionals (managers and administrators, professionals and para-professionals), white collar employees (clerks, sales-persons and personal service workers), and blue-collar employees (tradespersons, plant and machine operators and drivers, and labourers and related workers). Non-employed respondents were classified as home duties, retired, permanently unable to work, unemployed, or not easily classifiable (student, other, or missing).

#### Annual household income

Participants selected their pre-tax household income from 13 categories. These were recoded into six categories: ≥A$130,000, A$129,999- A$72,800, A$72,799-A$52,000, A$51,999-A$26,000, ≤A$25,999, and ‘don’t know’/ ‘don’t want to answer’.

#### Neighbourhood disadvantage

A neighbourhood socioeconomic disadvantage measure was derived using scores from the Australian Bureau of Statistics (ABS) Index of Relative Socioeconomic Disadvantage (IRSD) [35]. IRSD scores were calculated using 2006 census data and derived by the ABS using principal component analysis [36]. A neighbourhood’s IRSD score is a measure of an area’s overall level of disadvantage. It was calculated using 17 variables that captured a wide range of socioeconomic attributes, including education, occupation, income, unemployment, household structure and household tenure. For analysis, the 200 sampled neighbourhoods were grouped into quintiles, with Q1 denoting the 20% (*n* = 40) least disadvantaged and Q5 the most disadvantaged 20% (n = 40) areas, relative to the whole of Brisbane.

Covariates were age and sex. Age was derived from self-reported date of birth and categorised into five groups: 42–46, 47–51, 52–56, 57–61, 62–67 years.

### Statistical analysis

Of 7866 residents who completed the 2009 survey, the 568 (15%) who changed their residential address after the 2007 data collection were excluded to reduce potential selection bias due to movers being influenced by unmeasured preferences related to both residential choice and MWB [37]. Another 162 were excluded because they were not the same household respondent as in Wave 1, which resulted in their education data, collected in 2007, not being relevant to the data collected in 2009, and 277 were excluded because they had not completed all WEMWBS items. Respondents with missing data on any individual-level predictor variable, except occupation, were excluded from analysis (*n* = 138). After excluding these respondents, 6721 individuals were available for analyses.

Two multilevel linear regression models were used to examine associations between individual-level SEP, neighbourhood disadvantage and MWB. MWB score was the outcome variable. The independent variables of interest in Model 1 were education, household income and occupation as measures of individual-level SEP, with adjustment for age and sex. Model 2 added to Model 1 neighbourhood disadvantage as an independent variable of interest. Preliminary analysis showed that results for Models 1 and 2 were similar for men and women; therefore, they were analysed together. A sensitivity analysis was conducted to determine if the results for Model 2 changed when a variable representing the years lived at the current address was added. The results did not change, and hence, the results of the sensitivity analysis are not presented. Cross-level interactions between individual SEP and neighbourhood disadvantage on MWB scores were also modelled (adjusted for the other SEP variables) to examine variation in the mean MWB score for education, occupation and household income, by level of neighbourhood disadvantage. Data were analysed using Stata 15.1 (StataCorp, College Station, Texas).

## Results

The mean MWB score in the sample was 50.7, ranging from 14 to 70, with a standard deviation of 8.1. MWB scores were lowest for respondents aged 40–46 years, those with the least education, those who were permanently unable to work, members of the lowest income households, and residents of the most disadvantaged neighbourhoods (see Table 1).

**Table 1:** Sociodemographic characteristics and mean mental well-being scores for respondents in the analytic sample

***N*** = 6721 individuals N = 200 neighbourhoods	N	%	Mental well-being score	
			Mean	95% CI
**Total sample**	6721	100.0	50.7	50.5, 50.9
**Sex**				
Male	2872	42.7	50.3	50.0, 50.6
Female	3849	57.3	51.0	50.8, 51.3
**Age (y)**				
40–46	1414	21.0	49.4	49.4, 50.2
47–51	1434	21.3	49.7	49.3, 50.1
52–56	1389	20.7	50.7	50.3, 51.2
57–61	1300	19.3	51.4	50.9, 51.8
62–70	1184	17.6	52.4	51.9, 52.9
**Education**				
Bachelor’s degree and above	2201	32.7	51.6	51.3, 51.9
Diploma/associate degree	772	11.5	51.3	50.8, 51.9
Certificate (trade/business)	1179	17.5	50.8	50.4, 51.3
No post-school qualification	2569	38.2	49.8	49.5, 50.1
**Occupation**				
Manager/professional	2187	32.5	51.4	51.1, 51.7
White collar	1349	20.0	50.2	49.8, 50.7
Blue collar	837	12.5	49.8	49.3, 50.3
Home duties	382	5.7	51.0	50.2, 51.8
Retired	811	12.1	52.4	51.8, 53.0
Permanently unable to work	147	2.2	43.6	42.0, 45.2
Unemployed	116	1.7	47.2	45.5, 48.9
Not easily classifiable	892	13.3	50.7	50.1, 51.3
**Annual household income**				
A$130,000+	1279	19.0	52.1	51.7, 52.5
A$72,800–129,999	1744	25.9	51.0	50.7, 51.4
A$52,000–72,799	947	14.1	50.0	49.6, 50.6
A$26,000–51,599	1201	17.9	50.3	49.8, 50.7
Less than A$25,999	736	10.9	48.5	47.8, 49.2
Don’t know/ don’t want to answer	814	12.1	51.4	50.4, 52.0
**Neighbourhood disadvantage**				
Q1 (Least disadvantaged)	1732	25.8	51.6	51.2, 51.9
Q2	1422	21.1	51.2	50.8, 51.6
Q3	1372	20.4	50.5	50.1, 50.9
Q4	1300	19.3	50.5	50.0, 50.9
Q5 (Most disadvantaged)	895	13.3	49.1	48.5, 49.7

*Note* y, years; Q, quartile

Results of modelling the associations between individual-level SEP, neighbourhood disadvantage and MWB are shown in Table 2. In Models 1 and 2, MWB scores were statistically significantly lower for respondents who had the least amount of education compared with the most education, were permanently unable to work or unemployed compared with being in manager/professional positions, or had annual household incomes of <A$72,800 compared with A$130,000+. These results were attenuated in Model 2, yet remained statistically significant. Model 2 also showed that respondents living in the most disadvantaged neighbourhoods had statistically significantly lower MWB scores compared with those in those living in the least disadvantaged neighbourhoods.

**Table 2:** Multilevel linear regression results for the association between individual socioeconomic position and neighbourhood disadvantage on mental well-being

N = 6721	Model 1	Model 2
**N = 200 neighbourhoods**	**β (95%CI)**	**β (95%CI)**
**Individual-level SEP**		
Education		
Bachelor’s degree and above^a^	Ref	Ref
Diploma/associate degree	0.03 (−0.63, 0.68)	0.04 (−0.62, 0.70)
Certificate (trade/business)	0.01 (−0.60, 0.60)	0.05 (−0.55, 0.65)
No post-school qualification	**−1.29 (−1.80, − 0.78)**	**−1.22 (−1.74, − 0.71)**
Occupation^b^		
Manager/professional^a^	Ref	Ref
White collar	−0.44 (− 1.02, 0.15)	− 0.43 (− 1.03, 0.15)
Blue collar	− 0.25 (− 0.95, 0.44)	−0.19 (− 0.89, 0.50)
Home duties	− 0.13 (− 1.04, 0.77)	−0.15 (− 1.05, 0.76)
Retired	0.73 (− 0.02, 1.49)	0.73 (− 0.03, 1.50)
Permanently unable to work	**−5.62 (−7.02, −4.23)**	**−5.50 (−6.90, −4.10)**
Unemployed	**−2.66 (−4.16, − 1.16)**	**−2.62 (−4.12, − 1.13)**
Annual household income		
A$130,000 +^a^	Ref	Ref
A$72,800–129,999	− **1.07 (− 1.65, − 0.49)**	**− 1.02 (− 1.60, − 0.43)**
A$52,000–72,799	**−2.36 (−3.05, − 1.68)**	**−2.25 (− 2.94, − 1.56)**
A$26,000–51,599	**−2.41 (−3.09, − 1.73)**	**−2.27 (− 2.96, − 1.58)**
Less than A$25,999	**−3.99 (− 4.80, − 3.18)**	**−3.77 (− 4.59, − 2.94)**
**Neighbourhood disadvantage**		
Q1 (Least disadvantaged)^a^		Ref
Q2		0.07 (− 0.51, 0.65)
Q3		− 0.28 (− 0.87, 0.31)
Q4		−0.26 (− 0.87, 0.34)
Q5 (Most disadvantaged)		**−0.96 (−1.66, − 0.28)**

Notes: Australian dollar (A$). Boldface indicates statistical significance (*p* < 0.05); Model 1: education, occupation, and income adjusted for age and sex; Model 2: neighbourhood disadvantage, education, occupation, and income adjusted for age, sex

^a^Reference category

^b^Not easily classified category included in analysis, but not reported in the table

**Fig. 1:**
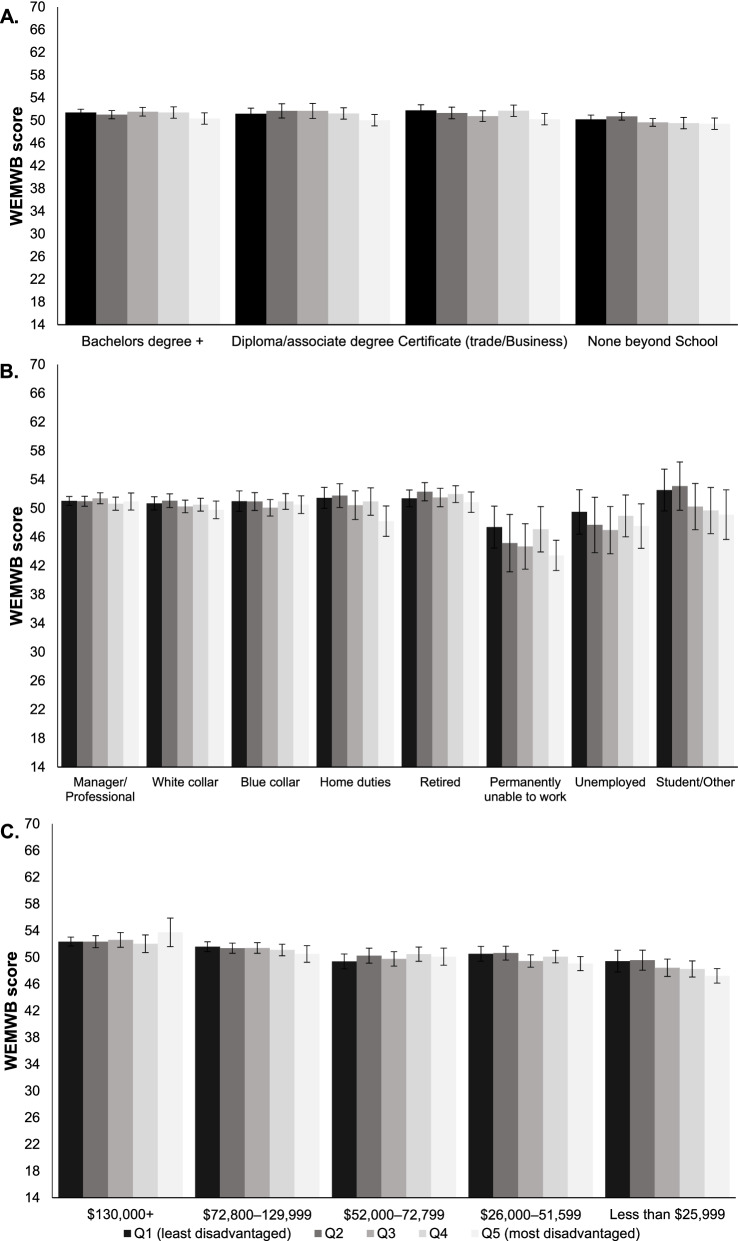
Predicted mean mental well-being by individual socioeconomic position and neighbourhood disadvantage

Cross-level interactions between individual-level SEP and neighbourhood disadvantage were not statistically significant (online Appendix Table 1). However, Fig. 1 suggested a ‘double disadvantage’ effect as evidenced by lower estimated MWB scores among respondents who were in the lowest categories of individual-level SEP (e.g., lowest levels of education, occupation and income) and living in the most disadvantaged neighbourhoods, relative to those of higher individual-level SEP and living in the least disadvantaged neighbourhoods.

## Discussion

This is the first known study to use multilevel analysis to examine associations between individual-level SEP (education, occupation and income), neighbourhood disadvantage and MWB, and their interactions. The results showed that both individual-level SEP and neighbourhood disadvantage, after adjusting for individual-level SEP, were associated with MWB. This finding suggests that the neighbourhood socioeconomic effects are important for MWB in addition to individual-level SEP effects.

This study’s findings at the individual-level are consistent with single-level studies of the UK population showing that adults with a low SEP, measured as education and occupation [6] or income, [15] have lower MWB. Despite limited research explaining how SEP contributes to MWB, researchers generally agree that education, occupation and income are conceptually distinct and contribute to health via different social processes through both separate [38] (e.g., education contributes directly to MWB) and linked pathways [22] (e.g., education contributes to MWB via income). For MWB, it has been suggested that intellectual, psychological, psychosocial, and material resources derive from education, occupation, and income, respectively [17, 20].

Earlier research examining associations between neighbourhood disadvantage and MWB is limited to two studies. Gale et al. [24] reported no association between area-level deprivation and MWB, a finding inconsistent with our study’s statistically significant results. The difference in results may be due to variation in the two studies’ samples and hence analytical techniques. Our sample included people from all strata of disadvantage and used a multilevel model, while Gale et al.’s sample had a low average level of area deprivation, due to the sample being derived from a socioeconomically advantaged county in the UK, necessitating the use of a single-level model. McAnerny et al. [23] presented unadjusted MWB means and confidence intervals for strata of neighbourhood disadvantage. Although linear patterning of unadjusted MWB scores and neighbourhood disadvantage in McAnerny’s et al. [23] and this study is similar, extrapolating relationships from biased results is problematic.

Results in this study are consistent with positive associations reported in the broader literature on the influences of neighbourhood on health-related outcomes [25]. The modest, but direct, neighbourhood disadvantage-MWB association suggests that the relationship was not fully confounded by education, occupation or income. Discerning theoretically meaningful pathways to explain what it is about neighbourhood disadvantage that contributes to MWB is challenging. Perhaps neighbourhood disadvantage, despite being an objective measure captured by 17 attributes, also represents mediating psychosocial factors that influence MWB [21]. Studies have highlighted several dimensions of MWB that are influenced by neighbourhood psychosocial factors. These include self-esteem arising from perceived neighbourhood attractiveness [19]; feelings of respect derived from how places are built, maintained, and respected by neighbours [19]; mental escape and attention restoration derived from green space [39]; and feelings of purpose, satisfaction, trust, reciprocity, and happiness arising from connectedness to community [22–24].

This is the first known study to examine cross-level interactions between individual-level SEP, neighbourhood disadvantage and MWB. Despite the interaction showing a pattern of ‘deprivation amplification’ when individual-level SEP and neighbourhood disadvantage interacted, this finding was not statistically significant. This finding supports McAneney et al.’s [23] findings about individual SEP and MWB; those researchers also did not find any interaction with education or income across five neighbourhood deprivation strata.

Several methodological limitations need to be taken into consideration when interpreting and generalising our results. First, causal inferences are limited by the cross-sectional design of the study. Although the plausibility of reverse causation between MWB and SEP indicators are unlikely, it is more likely for participants categorised as permanently unable to work. These participants may be permanently unable to work due to poor MWB. Second, the non-response rate for baseline 2007 HABITAT survey was 31.5%, and higher among those of low SEP and residents of disadvantaged neighbourhoods, a finding corroborated by prior studies examining participation rates of low SEP in survey research [38, 40]. Therefore, the response to the 2007 survey was likely to result in underestimation of socioeconomic variation in MWB. However, the minimal sample attrition between the 2007 and 2009 surveys did not affect generalisability, and analysis, not presented here, shows similar distribution of SEP characteristics between 2007 and 2009 surveys [31]. Third, the study and analytic design defined a neighbourhood as being synonymous with a CCD. Although spatial units (e.g., CCD) are used in most studies to define areas, there is a potential lack of intrinsic meaning to the neighbourhood context, unlike in studies that define areas as a delimiting radius around an individual’s residential location [41]. Fourth, the association between neighbourhood disadvantage and MWB may be confounded by unobserved individual-level socioeconomic factors. However, we adjusted our analyses for the three most commonly used individual-level measures of SEP in health research (i.e., education, occupation and income) [38, 42]. Fifth, our use of an index of disadvantage (IRSD) provided no direct assessment of a neighbourhood’s physical or social features that are amenable to change and may mediate MWB. However, evidence from studies of the built and social environments show that disadvantaged neighbourhoods in Brisbane have greater perceived and objective crime [43, 44]; greater exposure to traffic [45] (and noise and air pollution); and less greenspaces [46] and tree cover [47]. Sixth, measures of MWB other than the WEMWBS may tap into different MWB components that could provide different results.

## Conclusions

Respondents of low SEP or residing in disadvantaged neighbourhoods had low MWB. The neighbourhood disadvantage-MWB association was weaker than the individual SEP-MWB association but still suggested a modest but important effect of neighbourhood disadvantage on MWB, supporting previous multilevel studies, which commonly find modest neighbourhood-level effects [48].

Future research should focus on the underlying mechanisms driving the associations between individual- and neighbourhood-level disadvantage and MWB so that policies and programs can be developed. Moving from cross-sectional to multilevel longitudinal study designs would enable changes in psychosocial characteristics associated with individual- and neighbourhood-level disadvantage and low MWB to be examined. To address methodological challenges of observational studies, such as endogeneity, dependency, exchangeability and structural confounding, [49] researchers should consider using analytic techniques, such as propensity score and inverse probability weighting methods, to improve the utility of observational studies. Additionally, studies assessing the effects of neighbourhood disadvantage on MWB could test social causation and social selection mechanisms by examining individuals’ MWB before or after moving either into or out of neighbourhoods.

Until research establishes the mediating factors on the pathways between individual- and neighbourhood-level disadvantage and low MWB, knowledge created in this study has limited applied application for developing programs or interventions that improve MWB at a population level. However, this study does inform the development of policies designed to minimise inequalities in MWB at the individual- and neighbourhood-level in several ways: by providing population MWB scores for a normally distributed Australian population that can be used as a benchmark to evaluate interventions, policies and programs; contributing evidence to debates on health indicator selection and development that is used for population health and well-being monitoring and reporting; and identifying population groups with low MWB.

## Supplementary Information

**Additional file 1: Appendix Table 1.** Predicted Mental well-being (MWB) mean from individual-level socioeconomic position and neighbourhood disadvantage cross-level interactions.

## Data Availability

HABITAT study material and collaboration documents are available at https://cur.org.au/project/habitat/. Applicants must submit a HABITAT Expression of Interest to the study’s Principal Chief Investigator: gavin.turrell@rmit.edu.au.

## Abbreviations

ABS: Australian Bureau of Statistics
ANZCO: Australian and New Zealand Classification of Occupations
CCD: Census Collector’s Districts
HABITAT: How Areas in Brisbane Influence healTh and AcTivity
IRSD: Index of Relative Socioeconomic Disadvantage
MWB: Mental Well-being
NHMRC: (Australian) National Health and Medical Research Committee
SEP: Socioeconomic Position
WEMWBS: Warwick Edinburgh Mental Well-Being Scale

## References

[1] Reijneveld SA, Schene AH (1998). Higher prevalence of mental disorders in socioeconomically deprived urban areas in the Netherlands: community or personal disadvantage. J Epidemiol Community Health.

[2] Fryers T, Melzer D, Jenkins R (2005). The distribution of the common mental disorders: social inequalities in Europe. Clin Pract Epidemiol Ment Health.

[3] Jokela M (2020). Neighborhoods, psychological distress, and the quest for causality. Curr Opin Psychol.

[4] National Mental Health Commission. Monitoring mental health and suicide prevention reform: National Report 2019. 2019.

[5] Keyes CL (2005). Mental illness and/or mental health? Investigating axioms of the complete state model of health. J Consult Clin Psychol.

[6] Stewart-Brown S, Samaraweera PC, Taggart F (2015). Socioeconomic gradients and mental health: implications for public health. Br J Psychiatry.

[7] Taggart F, Stewart-Brown S, Parkinson J (2016). Warwick-Edinburgh mental well-being scale (WEMWBS) user guide - version 2.

[8] Chida Y, Steptoe A (2008). Positive psychological well-being and mortality: a quantitative review of prospective observational studies. Psychosom Med.

[9] Howell RT, Kern ML, Lyubomirsky S (2007). Health benefits: meta-analytically determining the impact of well-being on objective health outcomes. Health Psychol Rev.

[10] Cooper R, Stafford M, Hardy R (2014). Physical capability and subsequent positive mental wellbeing in older people: findings from five HALCyon cohorts. Age (Dordr).

[11] Rai R, Jongenelis M, Pettigrew S (2019). Identifying modifiable factors associated with health optimism in older adults. Aging Ment Health.

[12] Pressman SD, Cohen S (2005). Does positive affect influence health?. Psychol Bull.

[13] Huppert FA (2009). Psychological well-being: evidence regarding its causes and consequences. Appl Psychol Health Well Being.

[14] Steptoe A, Dockray S, Wardle J (2009). Positive affect and psychobiological processes relevant to health. J Pers.

[15] Ng Fat L, Scholes S, Boniface S (2017). Evaluating and establishing national norms for mental wellbeing using the short Warwick-Edinburgh mental well-being scale (SWEMWBS): findings from the health survey for England. Qual Life Res.

[16] Oakes JM, Rossi PH (2003). The measurement of SES in health research: current practice and steps toward a new approach. Soc Sci Med.

[17] Winkleby MA, Jatulis DE, Frank E (1992). Socioeconomic status and health: how education, income, and occupation contribute to risk factors for cardiovascular disease. Am J Public Health.

[18] Marmot M, Ryff CD, Bumpass LL (1997). Social inequalities in health: next questions and converging evidence. Soc Sci Med.

[19] Bond L, Kearns A, Mason P (2012). Exploring the relationships between housing, neighbourhoods and mental wellbeing for residents of deprived areas. BMC Public Health.

[20] Pinquart M, Sörensen S (2000). Influences of socioeconomic status, social network, and competence on subjective well-being in later life: a meta-analysis. Psychol Aging.

[21] Pickett KE, Pearl M (2001). Multilevel analyses of neighbourhood socioeconomic context and health outcomes: a critical review. J Epidemiol Community Health.

[22] Turrell G, Sanders AE, Slade GD (2007). The independent contribution of neighborhood disadvantage and individual-level socioeconomic position to self-reported oral health: a multilevel analysis. Community Dent Oral Epidemiol.

[23] McAneney H, Tully MA, Hunter RF (2015). Individual factors and perceived community characteristics in relation to mental health and mental well-being. BMC Public Health.

[24] Gale CR, Dennison EM, Cooper C (2011). Neighbourhood environment and positive mental health in older people: the Hertfordshire cohort study. Health Place.

[25] Diez Roux AV, Mair C (2010). Neighborhoods and health. Ann N Y Acad Sci.

[26] Turrell G, Lynch JW, Leite C (2007). Socioeconomic disadvantage in childhood and across the life course and all-cause mortality and physical function in adulthood: evidence from the Alameda County study. J Epidemiol Community Health.

[27] Huisman M, Kunst AE, Andersen O (2004). Socioeconomic inequalities in mortality among elderly people in 11 European populations. J Epidemiol Community Health.

[28] Macintyre S, Ellaway A, Cummins S (2002). Place effects on health: how can we conceptualise, operationalise and measure them. Soc Sci Med.

[29] Schüle SA, Bolte G (2015). Interactive and independent associations between the socioeconomic and objective built environment on the neighbourhood level and individual health: a systematic review of multilevel studies. PLoS One.

[30] Burton NW, Haynes M, Wilson LA (2009). HABITAT: a longitudinal multilevel study of physical activity change in mid-aged adults. BMC Public Health.

[31] Turrell G, Nathan A, Burton NW (2021). Cohort profile: HABITAT-a longitudinal multilevel study of physical activity, sedentary behaviour and health and functioning in mid-to-late adulthood. Int J Epidemiol.

[32] Dillman DA (2000). Mail and internet surveys: the tailored design method.

[33] Turrell G, Haynes M, Burton NW (2010). Neighborhood disadvantage and physical activity: baseline results from the HABITAT multilevel longitudinal study. Ann Epidemiol.

[34] Tennant R, Hiller L, Fishwick R (2007). The Warwick-Edinburgh mental well-being scale (WEMWBS): development and UK validation. Health Qual Life Outcomes.

[35] Australian Bureau of Statistics. 1220.0 - ANZSCO -- Australian and New Zealand Standard Classification of Occupations, 2013, Version 1.2. 2013.

[36] Rachele JN, Kavanagh AM, Badland H (2015). Associations between individual socioeconomic position, neighbourhood disadvantage and transport mode: baseline results from the HABITAT multilevel study. J Epidemiol Community Health.

[37] Hirsch JA, Moore KA, Clarke PJ (2014). Changes in the built environment and changes in the amount of walking over time: longitudinal results from the multi-ethnic study of atherosclerosis. Am J Epidemiol.

[38] Turrell G, Hewitt B, Patterson C (2003). Measuring socioeconomic position in dietary research: is choice of socioeconomic indicator important. Public Health Nutr.

[39] Houlden V, Weich S, Jarvis S (2017). A cross-sectional analysis of green space prevalence and mental wellbeing in England. BMC Public Health.

[40] Kavanagh AM, Goller JL, King T (2005). Urban area disadvantage and physical activity: a multilevel study in Melbourne, Australia. J Epidemiol Community Health.

[41] Riva M, Gauvin L, Barnett TA (2007). Toward the next generation of research into small area effects on health: a synthesis of multilevel investigations published since July 1998. J Epidemiol Community Health.

[42] Galobardes B, Lynch J, Smith GD (2007). Measuring socioeconomic position in health research. Br Med Bull.

[43] Foster S, Hooper P, Burton NW (2021). Safe habitats: does the association between neighborhood crime and walking differ by neighborhood disadvantage. Environ Behav.

[44] Fay-Ramirez S (2015). The comparative context of collective efficacy: understanding neighbourhood disorganisation and willingness to intervene in Seattle and Brisbane. Aust N Z J Criminol.

[45] Rachele JN, Learnihan V, Badland HM (2017). Neighbourhood socioeconomic and transport disadvantage: the potential to reduce social inequities in health through transport. J Transp Health.

[46] Astell-Burt T, Feng X, Mavoa S (2014). Do low-income neighbourhoods have the least green space? A cross-sectional study of Australia’s most populous cities. BMC Public Health.

[47] Shanahan DF, Lin BB, Gaston KJ (2014). Socioeconomic inequalities in access to nature on public and private lands: a case study from Brisbane, Australia. Landsc Urban Plan.

[48] Leyland AH, Groenewegen PP (2020). Multilevel Modelling for public health and health services research: health in context.

[49] Oakes JM, Andrade KE, Biyoow IM (2015). Twenty years of neighborhood effect research: an assessment. Curr Epidemiol Rep.

