# Correction to: Individual socioeconomic position, neighbourhood disadvantage and mental well-being: a cross-sectional multilevel analysis of mid-age adults

**DOI:** 10.1186/s12889-022-13084-1

**Published:** 2022-04-16

**Authors:** Emily M. Mann, Kristiann C. Heesch, Jerome N. Rachele, Nicola W. Burton, Gavin Turrell

**Affiliations:** 1 School of Public Health and Social Work, Queensland University of Technology, Victoria Park Road, Kelvin Grove QLD, Brisbane, Queensland 4059 Australia; 2 College of Health and Biomedicine and Institute for Health and Sport, Victoria University, Footscray, Victoria Australia; 3 School of Applied Psychology, Grifth University, Brisbane, Queensland Australia; 4 Menzies Health Institute Queensland, Grifth University, Brisbane, Queensland Australia; 5 School of Global, Urban and Social Studies, RMIT University, Melbourne, Victoria Australia

**Correction to: BMC Public Health 22, 494 (2022)**

**https://doi.org/10.1186/s12889-022-12905-7**

The original publication of this article contained 2 typo’s in the abstract. The incorrect and correct information is shown in this correction article [1]. The original article has been updated.

**Incorrect**

- This study **aims** to explore the relationship between individual socioeconomic position (SEP), neighbourhood disadvantage and mental well-being in mid-age adults.
- Multilevel modelling was used to analyse data collected from 7866 participants from the second (2009) wave of HABITAT (How Areas in Brisbane Influence healTh and activiTy), a longitudinal study (**2007–2018**) of adults aged 40–65 years living in Brisbane, Australia. Mental well-being was measured using the Warwick Edinburgh Mental Well-Being Scale (WEMWBS).

**Correct**

- This study **aimed** to explore the relationship between individual socioeconomic position (SEP), neighbourhood disadvantage and mental well-being in mid-age adults.
- Multilevel modelling was used to analyse data collected from 7866 participants from the second (2009) wave of HABITAT (How Areas in Brisbane Influence healTh and activiTy), a longitudinal study (**2007–2016**) of adults aged 40–65 years living in Brisbane, Australia. Mental well-being was measured using the Warwick Edinburgh Mental Well-Being Scale (WEMWBS).
