# Supplementary material for: Individual socioeconomic position, neighbourhood disadvantage and mental well-being: a cross-sectional multilevel analysis of mid-age adults
Source: BMC Public Health. 2022 Mar 14;22:494. doi: 10.1186/s12889-022-12905-7 (PMC8919596; doi:10.1186/s12889-022-12905-7)
Supplement: Supplementary file 1 — Additional file 1: Appendix Table 1. Predicted Mental well-being (MWB) mean from individual-level socioeconomic position and neighbourhood disadvantage cross-level interactions. [file 12889_2022_12905_MOESM1_ESM.docx]

Appendix Table 1. Predicted Mental well-being (MWB) mean from individual-level socioeconomic position and neighbourhood disadvantage cross-level interactions

|  | **Neighbourhood disadvantage** | | | | |
| --- | --- | --- | --- | --- | --- |
|  | **Q1 (least disadvantaged)** | **Q2** | **Q3** | **Q4** | **Q5 (most disadvantaged)** |
|  | **MWB mean (95% CI)** | **MWB mean (95% CI)** | **MWB mean (95% CI)** | **MWB mean (95% CI)** | **MWB mean (95% CI)** |
| **Individual-level SEP** |  |  |  |  |  |
| Education^a^ |  |  |  |  |  |
| Bachelor’s degree and above | 51.40 (50.82, 51.98) | 51.02 (50.30, 51.74) | 51.55 (50.77, 52.32) | 51.41 (50.51, 52.30) | 50.35 (49.12, 51.57) |
| Diploma/associate degree | 51.19 (50.19, 52.18) | 51.69 (50.44, 52.95) | 51.69 (50.36, 53.01) | 51.24 (50.02, 52.46) | 50.05 (48.32, 51.79) |
| Certificate (trade/business) | 51.77 (50.76, 52.78) | 51.33 (50.31, 52.36) | 50.76 (49.81, 51.72) | 51.71 (50.75, 52.67) | 50.24 (49.08, 51.39) |
| None beyond School | 50.19 (49.44, 50.95) | 50.74 (50.06, 51.41) | 49.66 (48.99, 50.33) | 49.54 (48.88, 50.20) | 49.43 (48.69, 50.17) |
| Occupation^b^ |  |  |  |  |  |
| Manager/professional | 51.02 (50.41, 51.64) | 50.98 (50.28, 51.68) | 51.39 (50.63, 52.16) | 50.64 (49.73, 51.56) | 50.94 (49.75, 52.13) |
| White collar | 50.69 (49.76, 51.61) | 51.06 (50.11, 52.01) | 50.26 (49.39, 51.14) | 50.50 (49.61, 51.39) | 49.78 (48.56, 51.00) |
| Blue collar | 50.99 (49.58, 52.41) | 50.94 (49.70, 52.18) | 50.07 (48.93, 51.22) | 50.94 (49.85, 52.04) | 50.50 (49.27, 51.74) |
| Home duties | 51.45 (49.99, 52.91) | 51.76 (50.10, 53.42) | 50.43 (48.43, 52.43) | 50.94 (49.03, 52.84) | 48.21 (46.10, 50.32) |
| Retired | 51.38 (50.21, 52.55) | 52.30 (50.04, 53.57) | 51.50 (50.21, 52.78) | 51.97 (50.80, 53.14) | 50.85 (49.42, 52.27) |
| Permanently unable to work | 47.38 (44.46, 50.30) | 45.15 (41.16, 49.14) | 44.69 (41.53, 47.85) | 47.08 (43.92, 50.23) | 43.45 (41.34, 45.56) |
| Unemployed | 49.50 (46.41, 52.59) | 47.69 (43.83, 51.54) | 46.96 (43.68, 50.24) | 48.94 (46.03, 51.86) | 47.53 (44.43, 50.62) |
| Annual household income^c^ |  |  |  |  |  |
| A$130,000+ | 52.36 (51.69, 53.03) | 52.34 (51.44, 53.25) | 52.61 (51.51, 53.72) | 52.03 (50.72, 53.35) | 53.74 (51.60, 55.88) |
| A$72,800–129,999 | 51.59 (50.85, 52.32) | 51.36 (50.60, 52.13) | 51.40 (50.61, 52.19) | 51.10 (50.24, 51.96) | 50.50 (49.25, 51.75) |
| A$52,000–72,799 | 49.39 (48.27, 50.50) | 50.25 (49.12, 51.37) | 49.76 (48.68, 50.84) | 50.47 (49.41, 51.53) | 50.10 (48.82, 51.38) |
| A$26,000–51,599 | 50.53 (49.41, 51.64) | 50.62 (49.59, 51.66) | 49.44 (48.52, 50.37) | 50.10 (49.17, 51.04) | 49.06 (48.00, 50.13) |
| Less than A$25,999 | 49.43 (47.79, 51.06) | 49.57 (48.07, 51.07) | 48.44 (47.14, 49.73) | 48.26 (47.05, 49.46) | 47.23 (46.14, 48.31) |
| Notes: Mental Well-Being (MWB); Australian dollar (A$)  Likelihood-ratio test results (format is Χ2 (df) = X, p = X):  ^a^ Χ^2^ (12) = 11.23, p = 0.50  ^b^ Χ^2^ (28) = 20.33, p = 0.85  ^c^ Χ^2^ (20) = 20.90, p = 0.64 | | | | | |
